# Microsatellite-based assessment of genetic diversity and inbreeding in the Ukrainian water buffalo (*Bubalus bubalis*) population

**DOI:** 10.14202/vetworld.2026.2133-2143

**Published:** 2026-05-21

**Authors:** Nataliia Mokhnachova, Kostiantyn Skrepets, Ostap Zhukorskyi, Lyubov Starodub, Volodymyr Ladyka, Olexander Tymchenko

**Affiliations:** 1 Institute of Animal Breeding and Genetics nd. a. M.V. Zubets of National Academy of Agrarian Sciences of Ukraine, Kyiv, Ukraine; 2 “Ascania Nova” Institute of Animal Breeding in the Steppe Regions named after M.F. Ivanov – National Scientific Selection-Genetics Center for Sheep Breeding, Kyiv, Ukraine; 3 Sumy National Agrarian University, Sumy, Ukraine

**Keywords:** allelic diversity, conservation genetics, genetic diversity, inbreeding, microsatellites, population structure, water buffalo, *Bubalus bubalis*

## Abstract

**Background and Aim:**

The Ukrainian water buffalo (*Bubalus bubalis*) represents a small, geographically isolated population with limited genetic characterization. Such demographic constraints increase the risk of reduced genetic variability and inbreeding, potentially compromising long-term sustainability. This study aimed to evaluate the genetic diversity and allelic structure of the Ukrainian water buffalo population using standardized microsatellite markers recommended by the Food and Agriculture Organization–International Society for Animal Genetics (FAO–ISAG).

**Materials and Methods:**

A total of 30 clinically healthy buffaloes from a major breeding nucleus in Ukraine were analyzed. Genomic DNA was extracted from whole blood samples and genotyped using seven short tandem repeat loci (BM1818, BM1824, BM2113, ETH10, ETH225, INRA023, and TGLA053). Allele frequencies and key population genetic parameters, including number of alleles (Na), effective number of alleles (Ne), observed heterozygosity (Ho), expected heterozygosity (He), polymorphism information content (PIC), and inbreeding coefficient (FIS), were calculated using GenAlEx software.

**Results:**

Five loci were polymorphic, while ETH10 and ETH225 were monomorphic. A total of 13 alleles were identified, with mean Na = 1.86 and Ne = 1.37. The average Ho (0.179) and He (0.196) indicated low genetic diversity within the population. INRA023 (He = 0.500; PIC = 0.375) and BM1824 (He = 0.413; PIC = 0.328) were the most informative markers. Most loci exhibited negative or near-zero FIS values, suggesting slight heterozygote excess; however, TGLA053 showed a high FIS (1.000), indicating heterozygote deficiency and potential inbreeding effects. Comparative analysis revealed substantially lower heterozygosity than other global buffalo populations, highlighting pronounced genetic differentiation.

**Conclusion:**

The Ukrainian water buffalo population demonstrates reduced genetic diversity, a limited allelic pool, and evidence of genetic isolation. These findings emphasize the urgent need for conservation strategies, including controlled introduction of unrelated breeding lines and continuous genetic monitoring using molecular markers. Such interventions are essential to enhance genetic variability, maintain adaptive potential, and ensure long-term population sustainability.

## INTRODUCTION

The domestic water buffalo (*Bubalus bubalis*) is an important livestock species that plays a significant role in maintaining food security, biodiversity, and sustainable agriculture in many countries around the world. This species is highly adaptable to different climatic conditions and is used for milk, meat, leather, and as a draft animal. However, despite its value, the genetic structure of many local buffalo populations has not yet been studied sufficiently, particularly the Ukrainian population [1, 2].

In Ukraine, the water buffalo population is small and isolated, which poses a risk of narrowing the gene pool, reducing genetic diversity, increasing inbreeding, and losing unique economically useful traits. To preserve the gene pool and plan effective breeding programs, modern methods of genetic monitoring are necessary, particularly molecular genetic analysis using microsatellite markers (STR markers), as recommended by FAO–ISAG for assessing genetic diversity in domestic animals [3–5].

Previous studies of microsatellite variability in buffaloes in different countries have shown significant differences in the level of genetic diversity depending on population size, history of formation, and selection direction. In buffalo populations of Asia, where large populations and a long history of domestication are preserved, a high level of polymorphism and heterozygosity has been reported. In contrast, European populations, formed from a limited number of ancestors and affected by genetic isolation, demonstrate reduced genetic variability. These findings [6–11] confirm that the intensity of selection processes, population size, and migratory connectivity between animal groups are key determinants of heterozygosity levels and gene pool structure. Understanding these patterns in a global context provides a foundation for evaluating the genetic status of the Ukrainian buffalo population and determining its position among other local populations of *B. bubalis*.

Microsatellite markers are an effective tool for assessing genetic diversity, heterozygosity, inbreeding, and familial relationships in domestic animal populations [12]. They enable detailed characterization of the allelic pool and the degree of genetic differentiation between animal groups, which is essential for conservation and breeding programs.

Despite the recognized importance of *B. bubalis* in global livestock production systems, there is a substantial lack of comprehensive genetic data on small and geographically isolated populations, particularly in Eastern Europe. Existing studies have predominantly focused on large populations in Asia and well-established herds in countries such as Italy, India, and Brazil, leaving critical knowledge gaps regarding the genetic structure, diversity, and inbreeding status of minor populations such as the Ukrainian water buffalo. Moreover, most available studies rely on broader population-level indicators without providing detailed locus-specific insights into allelic composition and heterozygosity patterns in such small populations. The absence of systematic genetic monitoring and limited application of standardized FAO–ISAG microsatellite panels further restricts the ability to compare these populations within a global framework. Consequently, there is insufficient evidence to guide effective conservation strategies, genetic improvement programs, and sustainable management practices for the Ukrainian buffalo population.

Therefore, the present study was designed to comprehensively evaluate the genetic diversity and allelic structure of the Ukrainian water buffalo population using standardized microsatellite (STR) markers recommended by FAO–ISAG. Specifically, the study aims to quantify allelic variation, assess observed and expected heterozygosity, estimate inbreeding coefficients, and identify the level of polymorphism within the population. In addition, the study seeks to compare these genetic parameters with those reported for other global buffalo populations to determine the relative genetic position and degree of differentiation of the Ukrainian population. The findings are expected to provide a robust scientific foundation for the development of targeted conservation strategies, genetic resource management, and sustainable breeding programs to enhance genetic variability and ensure the long-term viability of this endangered local population.

## MATERIALS AND METHODS

### Ethical approval

All experimental procedures involving animals were conducted in strict accordance with the ethical standards and guidelines for the care and use of animals in scientific research established by the National Academy of Agrarian Sciences of Ukraine (NAAS). The study protocol was reviewed and approved by the Commission on the Ethics of Animal Experiments of the Institute of Animal Breeding and Genetics named after M. V. Zubets, NAAS (Protocol No. 5, dated May 16, 2025).

The study complied with the principles outlined in the European Convention for the Protection of Vertebrate Animals Used for Experimental and Other Scientific Purposes (ETS No. 123) and adhered to internationally accepted animal welfare guidelines. All procedures were designed to minimize animal discomfort, distress, and physiological stress throughout the sampling process.

Blood sampling was performed by licensed veterinarians using standard aseptic techniques and low-stress handling procedures. Animals were gently restrained only for the minimal duration required for venipuncture, and no sedation or invasive interventions beyond routine blood collection were applied. All efforts were made to ensure animal welfare, including appropriate handling, avoidance of repeated sampling, and immediate release of animals following sample collection.

The animals included in this study were privately owned by the farm LLC “TASBIO” (Chernihiv region, Ukraine), and permission for sample collection and use in research was obtained from the farm management prior to the commencement of the study. No endangered or protected species were involved, and the study did not include experimental treatments, clinical interventions, or *in vivo* manipulations beyond routine sampling.

All biological samples were collected, transported, stored, and processed in compliance with institutional biosafety regulations. Data generated from this study were handled confidentially and used solely for scientific research purposes aimed at conservation genetics and sustainable breeding management of the Ukrainian water buffalo population.

### Study period and location

The study was conducted in 2024 at the Institute of Animal Breeding and Genetics named after M. V. Zubets, NAAS (Chubynske village, Kyiv region, Ukraine). The object of the study was the Ukrainian population of water buffaloes (*Bubalus bubalis*), which are maintained at the farm of LLC “TASBIO” (Chernihiv region, Ukraine).

**Figure 1:**
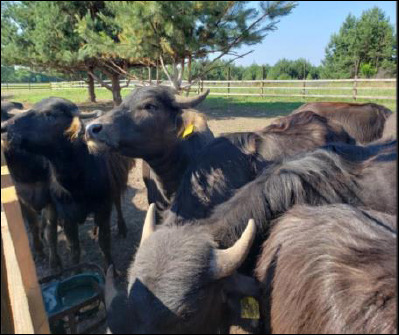
Photo of water buffaloes (*Bubalus bubalis*) at LLC “TASBIO” (Chernihiv region, Ukraine).

The selection of biological material was carried out from clinically healthy animals (Figure 1). The sampled group primarily consisted of animals from the lactating (milking) herd, reflecting the demographic structure of the farm and the practical management conditions at the time of sampling. Most sampled individuals were adult females actively involved in milk production, while a smaller proportion of males and non-lactating animals were included when available. The age distribution covered the productive age range (2–10 years), corresponding to the core breeding and milking population of the herd.

At the time of sampling, the herd maintained at LLC “TASBIO” comprised approximately 85 water buffaloes (farm records/official farm information), and thus the studied sample of 30 animals represented about 35% of the farm herd. According to available published data, the total national population of water buffaloes in Ukraine is estimated at approximately 120 animals; therefore, the TASBIO herd constitutes a major managed nucleus (about 71% of the national population), which is important when interpreting genetic drift and inbreeding risks in this small and isolated population.

### Study design

This study was designed as a cross-sectional molecular genetic analysis aimed at evaluating genetic diversity and population structure in the Ukrainian water buffalo population using microsatellite (STR) markers recommended by FAO–ISAG. A stratified sampling strategy was applied to ensure representation of both sexes and a broad age range (2–10 years). The study workflow included sample collection, genomic DNA extraction, polymerase chain reaction (PCR) amplification of selected microsatellite loci, electrophoretic separation of amplified products, allele scoring, and statistical genetic analysis.

### Sampling and DNA extraction

For molecular genetic analysis, whole blood samples from 30 buffaloes were selected. Animals were chosen using a stratified approach to include both sexes and a broad age range (2–10 years). Blood was collected from the jugular vein into tubes containing anticoagulant ethylenediaminetetraacetic acid in compliance with bioethical requirements and with minimal animal stress.

The samples were transported in a cooled state (4°C) to the laboratory of molecular genetics of the Institute of Animal Breeding and Genetics named after M. V. Zubets, NAAS, where they were stored at −20°C until further analysis.

Genomic DNA was isolated using a commercial kit DNA-sorb B (Amplisens, Moscow, Russia) according to the manufacturer’s instructions. The concentration and purity of DNA were assessed spectrophotometrically using NanoDrop ND-1000 (Thermo Scientific, Waltham, MA, USA) based on absorbance ratios at 260/280 nm. DNA quality was further evaluated using absorbance ratios at 260/280 nm and 260/230 nm. Only samples with A260/A280 ratios between 1.8 and 2.0 and A260/A230 ratios above 1.8 were considered suitable for downstream analysis.

DNA concentrations were normalized to approximately 20–50 ng/µL before PCR amplification. Extracted DNA was aliquoted to avoid repeated freeze–thaw cycles and stored at −80°C for long-term preservation, while working aliquots were maintained at −20°C for routine molecular analyses.

### Selection of microsatellite markers and DNA amplification

To analyze the genetic structure, seven microsatellite loci were used: BM1818, BM1824, BM2113, ETH10, ETH225, INRA023, and TGLA053 (Table 1). Primers were synthesized by Sigma (St. Louis, MO, USA) based on previously published sequences (Table 1). PCR cycling conditions were optimized for each locus, and all reactions were performed under identical thermal profiles to minimize inter-run variability.

**Table 1:** Nucleotide primer sequences for microsatellite loci.

No.	Microsatellite loci	Primer (5’–3’)	Annealing (°C)	Amplicons (bp)
1	BM1824 (chromosome 1)	gagcaaggtgtttttccaatc; cattctccaactgcttccttg	56	176–197
2	BM2113 (chromosome 2)	gctgccttctaccaaataccc; cttcctgagagaagcaacacc	58	122–156
3	INRA023 (chromosome 3)	gagtagagctacaagataaacttc; taactacagggtgttagatgaactc	58	195–225
4	ETH10 (chromosome 5)	gttcaggactggccctgctaaca; cctccagcccactttctcttctc	62	207–231
5	ETH225 (chromosome 9)	gatcaccttgccactatttcct; acatgacagccagctgctact	58	131–159
6	TGLA053 (chromosome 16)	gctttcagaaatagtttgcattca; atcttcacatgatattacagcaga	58	143–191
7	BM1818 (chromosome 23)	agctgggaatataaccaaagg; agtgctttcaaggtccatgc	58	248–278

The selection of microsatellite loci was based on FAO–ISAG recommendations for assessing genetic diversity in livestock populations. The panel of seven STR loci used in this study meets the minimum recommended set for initial characterization of genetic variability and allows comparison with previously published studies of buffalo populations using standardized markers.

A larger number of loci would increase the statistical power and resolution of diversity estimates. However, given the exploratory nature of this study, the selected panel was considered suitable for an initial assessment of the genetic status of the Ukrainian water buffalo population. Therefore, the results should be interpreted as a baseline genetic characterization rather than a comprehensive genome-wide analysis. Future studies should expand the marker panel to ≥20 microsatellite loci or incorporate high-density single nucleotide polymorphism (SNP) genotyping to provide more robust estimates of genetic diversity, population structure, and inbreeding.

### PCR amplification and electrophoresis

PCR was performed in a volume of 25 µL containing 50 ng of genomic DNA, 1× PCR buffer, 2 mM MgCl_2_, 200 µM of each deoxynucleotide triphosphate, 10 pmol of each primer, and 1 U of Taq polymerase (Thermo Scientific, Waltham, MA, USA). Amplification was carried out using an Applied Biosystems thermal cycler (Thermo Fisher Scientific) under the following conditions: initial denaturation at 94°C for 3 min, followed by 35 cycles of denaturation at 94°C for 30 s, annealing at 56–62°C for 30 s, elongation at 72°C for 30 s, and a final elongation step at 72°C for 10 min [13].

Amplification products were separated by polyacrylamide gel electrophoresis (5%–12%) and visualized under ultraviolet light after staining with ethidium bromide. In cases of scoring discrepancies, PCR amplification and electrophoresis were repeated to confirm allele identity and ensure consistency of genotyping results. Molecular weight markers pUC19 and Orange Ruler 20 bp (Thermo Scientific) were used to determine allele size.

PCR reactions included both positive and negative controls in each run. A known heterozygous DNA sample was used as a positive control, while a no-template control was included to detect contamination. All loci were amplified in singleplex reactions. Amplifications producing ambiguous banding patterns were repeated in duplicate to ensure reproducibility.

### Genetic data analysis

Allele frequencies for each locus were determined based on electrophoresis results. Genetic analyses were performed using GenAlEx version 6.503 implemented in Microsoft Excel. Standard population genetic parameters, including number of alleles (Na), observed heterozygosity (Ho), expected heterozygosity (He), and fixation indices (FIS), were calculated.

Hardy–Weinberg equilibrium was assessed using chi-square and exact tests implemented in GenAlEx. Linkage disequilibrium was evaluated pairwise among polymorphic loci [14]. Alleles were scored manually based on gel electrophoresis images and grouped within a 2 bp size interval. Rare alleles (frequency < 5%) were verified through repeated amplification and electrophoresis.

Due to the relatively small sample size and limited polymorphism observed in several loci, extended population genetic analyses were not applied. However, such analyses are recommended for future monitoring and conservation-oriented genetic studies. Criteria recommended by FAO [15] were used to assess polymorphism and genetic diversity levels.

The overall workflow of the study included the following steps: blood collection → genomic DNA extraction → PCR amplification of microsatellite loci → polyacrylamide gel electrophoresis → allele scoring and validation → statistical genetic analysis using GenAlEx software.

## RESULTS

### Allelic diversity and microsatellite polymorphism

To characterize the genetic structure of the Ukrainian population of water buffaloes (*Bubalus bubalis*), seven microsatellite loci were analyzed: BM1818, BM1824, BM2113, ETH10, ETH225, INRA023, and TGLA053. Five loci (BM1818, BM1824, BM2113, INRA023, and TGLA053) were polymorphic, whereas ETH10 and ETH225 were monomorphic. A total of 13 alleles were identified in the population. The number of alleles per locus (Na) ranged from 1 to 3, with an average value of Na = 1.857, and the effective number of alleles (Ne) varied from 1.000 to 2.000 (Table 2).

**Table 2:** Genetic structure of the Ukrainian water buffalo (*Bubalus bubalis*) population based on allele frequency of microsatellite loci.

Locus	Allele	Allele frequency	Na	Ne
BM1818	256	0.042	3	1.291
	268	0.875		
	274	0.083		
BM1824	188	0.708	2	1.704
	202	0.292		
BM2113	129	0.958	2	1.087
	133	0.042		
ETH10	209	1.000	1	1.000
ETH225	140	1.000	1	1.000
INRA023	206	0.500	2	2.000
	208	0.500		
TGLA053	145	0.875	2	1.280
	147	0.125		

The greatest allelic diversity was observed at the BM1818 locus, where three alleles (256, 268, and 274) were identified; allele 268 was the most frequent (0.875). At the BM1824 locus, two alleles were detected (188 with a frequency of 0.708 and 202 with a frequency of 0.292). The BM2113 locus was characterized by the predominance of allele 129 (0.958), whereas allele 133 was rare (0.042).

**Figure 2:**
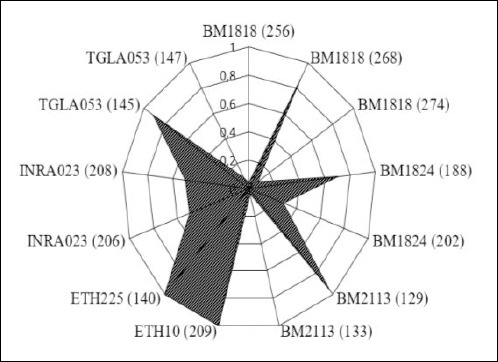
Distribution of alleles of the analyzed microsatellite loci.

The INRA023 locus showed a balanced distribution of two alleles (206 and 208), each with a frequency of 0.500, indicating equal representation within the population. The TGLA053 locus also contained two alleles (145 and 147), with a marked predominance of allele 145 (0.875). In contrast, ETH10 and ETH225 were monomorphic, exhibiting only alleles 209 and 140, respectively (Figure 2).

### Heterozygosity, polymorphism, and FIS

A more comprehensive understanding of population genetic structure requires the integration of multiple analytical parameters reflecting genetic variability. The analysis of the allele pool across seven STR loci provided detailed insights into the polymorphism and heterozygosity of the studied population. The results of this assessment are presented in Table 3.

**Table 3:** Parameters of the genetic structure of the Ukrainian water buffalo population based on STR loci.

Locus	Ho	He	PIC	FIS
BM1818	0.250	0.226	0.212	−0.108
BM1824	0.417	0.413	0.328	−0.008
BM2113	0.083	0.080	0.077	−0.043
ETH10	0.000	0.000	0.000	–
ETH225	0.000	0.000	0.000	–
INRA023	0.500	0.500	0.375	0.000
TGLA053	0.000	0.153	0.141	1.000

Ho = observed heterozygosity, He = expected heterozygosity, PIC = polymorphism information content, FIS = Wright’s fixation index.

The observed heterozygosity (Ho) ranged from 0.000 to 0.500, while the expected heterozygosity (He) varied within the same range. The mean He value across all loci was 0.196, indicating low genetic diversity based on microsatellite analysis. The polymorphism information content (PIC) ranged from 0.000 to 0.375 and was low for most loci (≤0.328), except for BM1824 (PIC = 0.328) and INRA023 (PIC = 0.375), which were relatively more informative for population genetic analysis.

Wright’s fixation index (FIS), reflecting the deficit or excess of observed heterozygosity relative to expected values, was negative or close to zero for most loci, indicating a slight excess of heterozygotes. The only exception was locus TGLA053, where the maximum possible FIS value (1.000) was observed due to the complete absence of heterozygotes.

### Hardy–Weinberg equilibrium and linkage disequilibrium

Hardy–Weinberg equilibrium testing revealed that deviations were locus-specific. A significant departure from equilibrium was detected at TGLA053, characterized by heterozygote deficiency despite non-zero expected heterozygosity. In contrast, the remaining polymorphic loci (BM1818, BM1824, BM2113, and INRA023) did not show consistent or statistically significant deviations after Bonferroni correction.

Pairwise linkage disequilibrium analysis did not reveal stable associations among loci, suggesting that the observed genetic patterns are not driven by genome-wide linkage effects.

### Interpretation of genetic structure and diversity

The absence of heterozygotes at locus TGLA053 may reflect demographic processes such as inbreeding and reduced effective population size. However, locus-specific factors, including the potential presence of null alleles, cannot be excluded. No comparable heterozygote deficiency patterns were observed at other loci, indicating that the extreme FIS value at TGLA053 should be interpreted cautiously, particularly given the sensitivity of single-locus estimates in small and genetically constrained populations.

Overall, the genetic analysis of microsatellite loci in the studied water buffalo population indicates a low level of genetic diversity. This is supported by the limited number of detected alleles, low polymorphism levels, and reduced heterozygosity values. These findings suggest constraints in the gene pool and potential effects of inbreeding, likely resulting from directional selection, geographic isolation, and small population size.

## DISCUSSION

### Population size, genetic drift, and heterozygosity patterns

Given that the total national population of Ukrainian water buffalo is estimated at approximately 120 individuals and that the studied herd represents the majority of the managed breeding nucleus, the reduced heterozygosity observed in this study is consistent with long-term demographic contraction and limited gene flow rather than solely a sampling artifact. In small and partially closed populations, even moderate relatedness among breeding animals may substantially reduce the effective population size (Ne), thereby accelerating genetic drift and contributing to the erosion of genetic diversity.

The deviation from Hardy–Weinberg equilibrium was primarily locus-specific (TGLA053) rather than uniform across all markers, suggesting that heterozygote deficiency may reflect a combination of demographic processes and marker-specific effects rather than generalized genome-wide inbreeding alone.

### Interpretation of clustering and analytical limitations

The clustering analysis based on Ho and He values should be interpreted as exploratory. Because Euclidean distances were derived from summary heterozygosity indices rather than full allele frequency matrices across comparative populations, the resulting dendrogram does not represent a formal reconstruction of genetic relationships and should not be considered primary evidence of population isolation.

For a deeper understanding of evolutionary processes, the formation history of the Ukrainian buffalo population, and its position among other populations, it is important to consider genetic distance or genetic similarity as key parameters in population genetics. Comparison with global buffalo populations enables the assessment of relatedness with geographically distant groups, clarification of genetic origin, and evaluation of evolutionary processes shaping population structure.

Such analyses are also of practical importance for planning gene pool conservation programs. Genetic distance is not only an indicator of evolutionary history but also an applied tool for biodiversity conservation and optimization of breeding strategies. Identification of genetically related or distant populations can guide the selection of breeding material and the development of conservation-oriented mating schemes.

The magnitude of genetic distance reflects differences in allelic composition and forms the basis for constructing phylogenetic trees and dendrograms illustrating population divergence, relatedness, and potential migration pathways. Classical approaches for estimating genetic distance, such as those described by [16–18], require allele frequency data for each locus. However, such data were not available in many of the reviewed studies, as allele frequencies were often not reported or the microsatellite panels did not overlap with those used in the present study.

### Comparative genetic diversity across global populations

Instead, most comparative studies report general indicators of genetic diversity, particularly observed heterozygosity (Ho) and expected heterozygosity (He) (Table 3) [19–23]. Therefore, the comparative framework based on Ho and He values should be considered indicative rather than definitive. More robust interpopulation inferences will require allele frequency–based metrics, such as fixation index (FST) or allele-sharing approaches, calculated using harmonized marker panels across populations.

**Figure 3:**
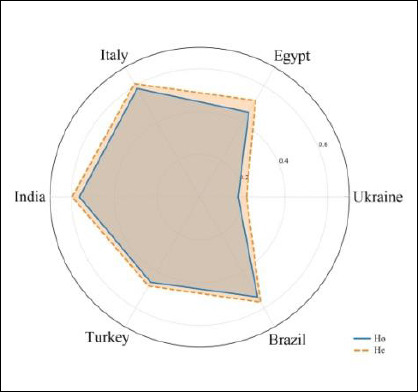
Diagram of average heterozygosity indicators based on microsatellite loci in water buffalo populations from different countries.

Table 4 and Figure 3 present the generalized average values of Ho and He for the Ukrainian water buffalo population compared with previously published data from other countries.

**Table 4:** Average observed and expected heterozygosity of water buffalo populations based on STR loci.

Country	Ho (average)	He (average)	Number of loci	Data source
Ukraine	0.179	0.196	7	Present study
Egypt	0.457	0.521	13	[18]
Italy	0.586	0.612	10	[19]
India	0.563	0.597	20	[20]
Turkey	0.460	0.480	10	[21]
Brazil	0.540	0.568	15	[22]

Ho = observed heterozygosity, He = expected heterozygosity , STR = short tandem repeat.

A comparative analysis of genetic diversity showed that buffalo populations from Egypt (Ho = 0.457; He = 0.521) [19] and Turkey (Ho = 0.460; He = 0.480) [22] exhibit similar levels of genetic diversity, both exceeding the Ukrainian population by more than twofold. The highest heterozygosity values were observed in Italian [20] and Indian [21] buffalo populations. Italian buffaloes demonstrated Ho = 0.586 and He = 0.612, reflecting a high level of genetic diversity maintained through large population size and structured breeding programs. Similarly, Indian buffaloes exhibited Ho = 0.563 and He = 0.597, indicative of a broad genetic base and active random mating processes.

The Brazilian population (Ho = 0.540; He = 0.568) [23] also showed high genetic diversity, comparable to Indian and Italian populations, likely due to the use of multiple breeding lines. In contrast, the Ukrainian buffalo population exhibited the lowest Ho (0.179) and He (0.196) values among all analyzed groups, indicating a substantial reduction in genetic diversity. This reduction is likely associated with small herd size, long-term use of a limited number of breeding animals, and increased inbreeding. These findings highlight the urgent need to expand the gene pool through controlled introduction of unrelated animals and the implementation of conservation programs.

### Genetic similarity and clustering analysis

Hierarchical clustering based on Euclidean distances was used to assess genetic similarity between populations using mean Ho and He values. This approach is suitable when allele frequency data are unavailable but general population genetic parameters are provided. Euclidean distance allows integration of multiple parameters into a single measure of divergence, facilitating visualization through dendrograms. Similar methodologies have been reported in population genetics studies [24–27]. The resulting values were used to construct a hierarchical dendrogram using the unweighted pair group method with arithmetic mean (Table 5).

**Table 5:** Genetic similarity indices of water buffalo populations from different countries.

Country	Ukraine	Egypt	Italy	India	Turkey
Egypt	0.390	–	–	–	–
Italy	0.479	0.137	–	–	–
India	0.458	0.113	0.032	–	–
Turkey	0.310	0.041	0.157	0.129	–
Brazil	0.431	0.069	0.049	0.029	0.100

The highest genetic similarity was observed between Egyptian and Turkish populations (distance 0.041), as well as between Indian and Italian populations (0.032), suggesting shared ancestry or historical gene flow. High similarity was also identified between Indian and Brazilian populations (0.029), consistent with documented historical introduction of Indian buffaloes into Latin America [28].

In contrast, the Ukrainian buffalo population exhibited the greatest genetic differentiation from all other groups (distance range 0.310–0.479), indicating population isolation, limited gene flow, and reduced genetic diversity. This distinctiveness underscores the need for targeted conservation strategies, including controlled introduction of unrelated breeding lines.

### Dendrogram interpretation and population structure

**Figure 4:**
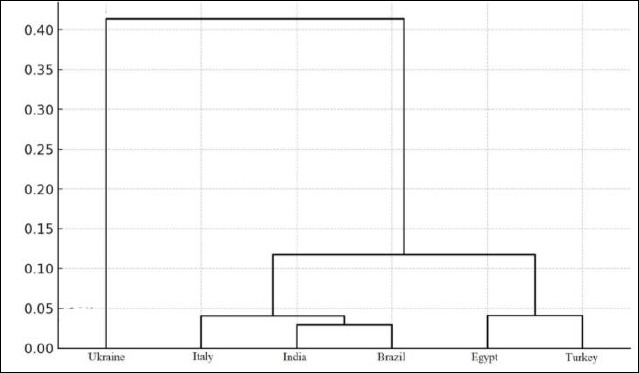
Dendrogram illustrating genetic similarity among water buffalo populations based on STR loci.

Genetic relationships among populations are further illustrated by hierarchical clustering and dendrogram construction (Figure 4). Euclidean distance was used as a measure of divergence, integrating Ho and He values. However, this clustering approach remains exploratory because it is based on summary indices rather than allele frequency matrices. Therefore, the dendrogram should be interpreted as supportive rather than definitive evidence of population relationships.

The smallest genetic distance was observed between Indian and Brazilian populations (0.029), forming a closely related cluster, likely reflecting shared ancestry and historical exchange of breeding material. Similar relationships were observed between Indian and Italian populations (0.032) and between Italian and Brazilian populations (0.049), forming a secondary cluster. Egyptian and Turkish populations formed a separate cluster (distance 0.041), consistent with geographic proximity and historical connections.

In contrast, the Ukrainian buffalo population showed clear separation from all other populations (distance 0.310–0.479), indicating long-term isolation and limited gene flow.

### Conservation implications and future strategies

The results confirm the distinct genetic structure of the Ukrainian water buffalo population and highlight the uniqueness of its gene pool. According to conservation genetics criteria, populations with an effective size exceeding 500 individuals are considered stable, those between 50 and 500 are vulnerable, and populations below 50 are at high risk of extinction [29].

Overall, the findings are consistent with previous studies showing that geographically isolated buffalo populations with small effective population sizes exhibit reduced heterozygosity and increased genetic differentiation [19, 20, 22].

Based on these results, practical conservation strategies should be implemented. Controlled introduction of new breeding lines should follow a structured genetic rescue approach rather than unsystematic crossbreeding. Selection of donor populations should be based on comparable genetic data obtained using harmonized marker panels (expanded microsatellite sets or SNP genotyping), estimates of genetic differentiation (e.g., FST or allele-sharing metrics), and availability of breeding material.

The introduction of unrelated individuals should be gradual (e.g., 2–4 animals per breeding cycle) to increase allelic diversity while maintaining population stability. These interventions must be accompanied by veterinary screening and quarantine procedures to prevent disease introduction.

Potential risks, including maladaptation and outbreeding depression, should be evaluated before implementation through assessment of ecological compatibility, reproductive performance, and genetic distance. Post-introduction monitoring should include repeated evaluation of heterozygosity, inbreeding coefficients (FIS), effective population size, and reproductive success over at least two breeding cycles. Controlled mating strategies should also be maintained to minimize close-kin mating and preserve rare alleles.

Such an evidence-based conservation approach would enable the Ukrainian water buffalo population to increase genetic variability while maintaining adaptive potential and long-term demographic stability.

## CONCLUSION

The present study provides a baseline molecular characterization of the Ukrainian water buffalo population using FAO–ISAG-recommended STR markers. The results demonstrated low genetic diversity, as reflected by a limited allelic pool (13 alleles across seven loci), low mean expected heterozygosity (He = 0.196), and low PIC for most loci. Two loci (ETH10 and ETH225) were monomorphic, whereas only five loci were polymorphic. The highest informativeness was observed for INRA023 (PIC = 0.375) and BM1824 (PIC = 0.328). Most loci showed negative or near-zero FIS, indicating a slight excess of heterozygotes; however, TGLA053 exhibited complete heterozygote deficiency (FIS = 1.000), suggesting locus-specific effects and potential inbreeding. Comparative analysis revealed that the Ukrainian population has substantially lower Ho and He values than populations from Egypt, Italy, India, Turkey, and Brazil, confirming pronounced genetic depletion and isolation.

From a practical standpoint, these findings highlight the urgent need for targeted conservation and breeding interventions. The controlled introduction of unrelated breeding individuals, guided by comparable genetic data and harmonized marker panels, is recommended to increase allelic diversity while minimizing disruption of the existing genetic structure. The implementation of structured mating schemes, routine genetic monitoring using STR or high-density SNP markers, and strict biosecurity protocols will be essential to prevent further loss of genetic variability and to sustain long-term productivity.

A key strength of this study lies in the use of standardized FAO–ISAG microsatellite markers, enabling comparison with global buffalo populations and providing a reliable initial assessment of genetic status. In addition, the relatively high sampling proportion (approximately 35% of the herd and a major fraction of the national population) enhances the representativeness of the findings for this small and isolated population.

However, several limitations should be acknowledged. The use of a limited number of loci restricts the resolution of genetic diversity estimates, and the absence of allele frequency data from comparative studies constrains robust interpopulation analyses. Furthermore, the small population size and concentration of animals within a single managed nucleus may introduce bias due to relatedness and genetic drift.

Future studies should expand the microsatellite panel (≥20 loci) or employ genome-wide SNP genotyping approaches to provide higher-resolution insights into population structure, genetic differentiation, and inbreeding dynamics. Longitudinal monitoring across multiple breeding cycles, coupled with the evaluation of reproductive performance and adaptability, will be critical to assess the effectiveness of conservation strategies and genetic rescue interventions.

In conclusion, the Ukrainian water buffalo population exhibits clear signs of reduced genetic diversity and genetic isolation, emphasizing its vulnerability and the need for immediate conservation action. The findings of this study provide a scientific foundation for evidence-based management strategies aimed at preserving this unique genetic resource while enhancing its long-term sustainability and adaptive potential.

## DATA AVAILABILITY

The data generated during the study are included in the manuscript.

## AUTHORS` CONTRIBUTIONS

NM: Conceptualized and designed the study. NM and KS: Conceptualization, methodology, data collection, formal analysis, and writing of the original draft. LS, OZ, VL, and OT: Investigation, data interpretation, manuscript review and editing, and supervision. All authors have read and approved the final manuscript.
